# Does Periodontal Tactile Input Uniquely Increase Cerebral Blood Flow in the Prefrontal Cortex?

**DOI:** 10.3390/brainsci10080482

**Published:** 2020-07-26

**Authors:** Takaharu Goto, Nobuaki Higaki, Takahiro Kishimoto, Yoritoki Tomotake, Tetsuo Ichikawa

**Affiliations:** 1 Department of Prosthodontics and Oral Rehabilitation, Tokushima University Graduate School of Biomedical Sciences, Tokushima 770-8504, Japan; nhigaki@bf6.so-net.ne.jp (N.H.); c301751010@tokushima-u.ac.jp (T.K.); ichi@tokushima-u.ac.jp (T.I.); 2 Oral Implant Center, Tokushima University Hospital, Tokushima University, Tokushima 770-8504, Japan; tomotake.dent@tokushima-u.ac.jp

**Keywords:** prefrontal cortex, functional near-infrared spectroscopy, periodontal tactile input, occlusal force

## Abstract

We previously studied the effect of peripheral sensory information from sensory periodontal ligament receptors on prefrontal cortex (PFC) activity. In the dental field, an alternative dental implant without periodontal sensation can be applied for missing teeth. In this study, we examine whether periodontal tactile input could increase cerebral blood flow (CBF) in the PFC against elderly patients with dental implants lacking periodontal tactile (implant group), elderly individuals with natural teeth (elderly group), and young individuals with natural teeth (young group). The experimental task of maintaining occlusal force as closed-loop stimulation was performed. Compared with the young group, the elderly group showed significantly lower CBF. Contrastingly, compared with the young group, the implant group showed significantly lower CBF. There were no significant differences between the elderly and implant groups. Regarding the mean occlusal force value, compared with the young group and the elderly group, the implant group had a numerically, but not significantly, larger occlusal force exceeding the directed range. In conclusion, the periodontal tactile input does not uniquely increase PFC activity. However, increased CBF in the PFC due to the periodontal tactile input in the posterior region requires existing attention behavior function in the PFC.

## 1. Introduction

The prefrontal cortex (PFC) is located in the anterior cerebral cortex and plays an important role in executive control, including complex cognitive behavioral planning, decision making, and social behaviors [1,2,3]. Considering the increasing elderly population, cognitive decline and dementia have become major worldwide problems [4]; moreover, the importance of sensory information for PFC activity has been highlighted. Specifically, regarding periodontal tactile sensation, several epidemiological studies have reported a relationship between tooth loss (loss of occlusal support) and cognitive decline/dementia [5,6,7]. Therefore, there is interest regarding the effect of periodontal tactile sensation on PFC activity and the underlying mechanisms.

Generally, it is difficult to examine the effect of peripheral sensory information on PFC activity with separation of somatosensory movement and sensation to determine the relationship between their presence or absence and PFC activity. However, periodontal tactile sensation is easily blocked through local anesthesia and can be easily separated from oral movements [8]. We previously studied the effect of peripheral sensory information from sensory periodontal ligament receptors on PFC activity [9,10]. This involved a task that controlled cognitive function involving maintaining a target occlusal force using closed-loop stimulation. Specifically, this was a feedback control task that used auditory and visual information. Among young individuals, we found that periodontal tactile input in molar teeth promoted PFC activity, which significantly downregulated occlusal force caused by the task.

Furthermore, in the dental field, an alternative dental implant without periodontal sensation can be applied for missing teeth where patients are similarly asked to maintain a target occlusal force [11,12,13]. Therefore, we employed the same experimental protocol among elderly patients with dental implants lacking periodontal tactile, elderly individuals with natural teeth, and young individuals with natural teeth to examine whether periodontal tactile input could increase cerebral blood flow (CBF) in the PFC.

## 2. Materials and Methods

### 2.1. Participants

We enrolled 18 participants from Tokushima University Hospital and divided them into three groups. The young group included six healthy Japanese participants (five males and one female; mean age: 26.0 ± 1.41 years) with normal teeth. The elderly group included six elderly participants (one male and five females; mean age: 69.8 ± 7.3 years) who were capable of occlusion with their natural upper and lower teeth. Participants in the aforementioned groups had a complete dental arch except for the third molar. The implant group included six elderly participants (three males and three females; mean age: 69.0 ± 7.2 years) who could achieve occlusion with their upper and lower dental implants and had complete dental arch except for the third molar. All participants lacked dental problems or complications of the central nervous system. All participants provided written informed consent after being briefed regarding the study’s objectives and methodology. Data were collected from each patient over one day, with data for young participants being collected from a previous report [9]. This study was approved by the ethics committee of Tokushima University Hospital (No. 1780), and all experiments were performed in accordance with relevant guidelines and regulations.

### 2.2. Experimental Apparatus

**Figure 1:**
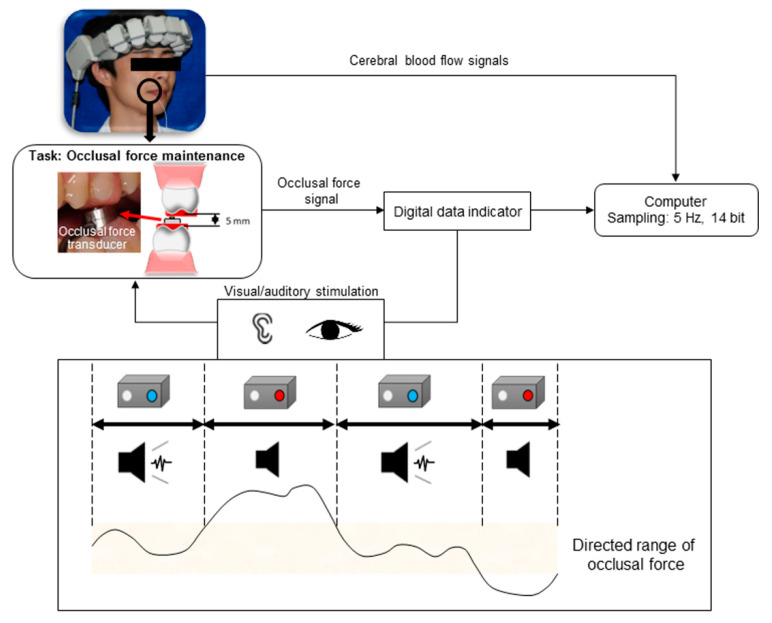
Experimental apparatus.

Figure 1 shows the experimental apparatus. PFC activation was evaluated in unrestrained participants using a wearable functional near-infrared spectroscopy (fNIRS) device (WOT-100, HITACHI, Tokyo, Japan). Ten measuring probes were spaced at 30 mm intervals where they covered three measurement areas around the frontal pole, including Broadmann’s area 10 (ch 1~3) and the dorsolateral PFC, the right side of Broadmann’s area 46 (ch 4~7), and the left side of Broadmann’s area 46 (ch 8~10). Relative changes in oxy-hemoglobin levels were measured using light attenuation at two wavelengths of 705 nm and 830 nm.

The occlusal force was measured using a commercial load cell (UNCDW-200N, Unipulse Corporation, Tokyo, Japan). This load cell, which is an occlusal force transducer, was modified using an auto-curing acrylic resin and fitted to the occlusal surface to accommodate the upper and lower first molar bites. The interocclusal distance was 5 mm. Signals from the occlusal force transducer were transferred to a computer via a digital data acquisition device (F372A, Unipulse Corporation, Tokyo, Japan) at 5 Hz frequency with 14-bit accuracy.

### 2.3. Task

The experimental task of maintaining occlusal force as closed-loop stimulation was performed as previously described [9,10]. Measurements were performed in a shielded soundproof room that eliminated external disturbances. There were minimal changes in room lighting and temperature. During measurements, the participants were seated on a chair in a relaxed state and were asked to continuously bite an occlusal force transducer at 27.5 ± 2.5 N (instructed range) for 30 s. The participants practiced maintaining the occlusal force for two minutes using visual and auditory indicators before the experimental task. However, the values displayed on the indicators were hidden during the experimental task.

Either visual or auditory guidance was employed to guide occlusal force maintenance. Green (with the buzzer off) and red (with the buzzer on) LED lights were illuminated when the measured occlusal force value was within or outside the reference range, respectively. During the tests, the participants performed occlusal force maintenance with visual and auditory guidance, as well as without external guidance, where the order of the three conditions was randomized. One measurement course consisted of the following three phases: rest without a task for 2 min, practice for 2 min, and measurement for 30 s. Each condition was repeated four times on separate days, and their mean value was obtained.

### 2.4. Statistical Analyses

Data obtained from fNIRS and the occlusal force transducer were synchronized for subsequent analyses. The mean CBF in the PFC during the first 10 s was defined as the baseline and used for data correction. The period between 10 and 30 s after task onset was analyzed to exclude early adjustments, including the rapid increase in force before achieving a constant occlusal force.

We calculated the mean values of CBF in the PFC and occlusal force, as well as the coefficient of variation, which indicated occlusal force stability. The coefficient of variation was calculated by dividing the standard deviation by the mean value. A one-way analysis of variance with Bonferroni post-hoc test was performed for between-group comparison of vertebral blood flow and occlusal force using SPSS^®^ version 25.0 software (IBM Corp. Armonk, NY, USA). We set statistical significance at a *p*-value of 5%.

## 3. Results

**Figure 2:**
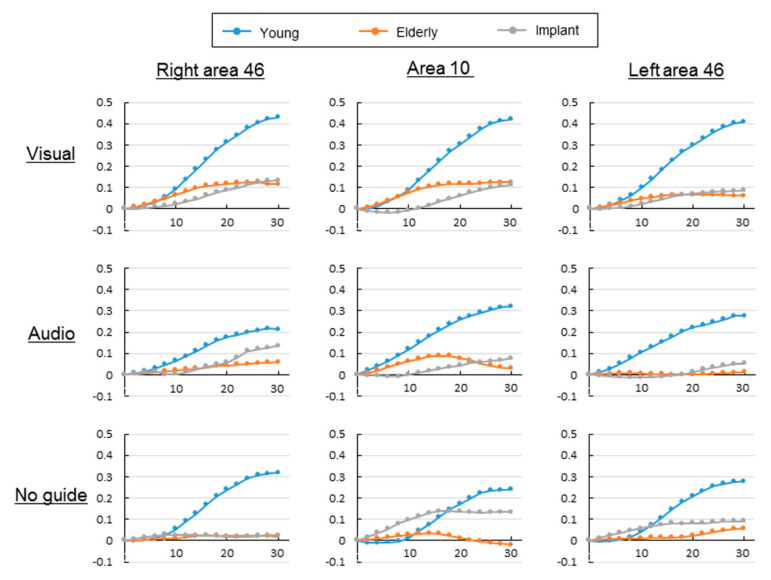
Change patterns in regional cerebral blood flow in the prefrontal cortex (CBF-PFC) in all three groups: visual, with visual information; audio, with auditory information; and no guide, without external information (unit of horizontal axis: seconds; unit of vertical axis: mM·mm).

Figure 2 presents the temporal patterns of the average CBF during the 30 s task in each condition. In the young group, there was a monotonic increase in the CBF after the onset of tasks involving visual and auditory information. In the task without external information, there was either no decrease or a slight decrease in CBF for a certain period after task onset; however, there was a subsequent tendency to increase. Compared with the young group, the elderly group showed a smaller tendency to increase, especially in the task with visual information. Moreover, the CBF increased until it plateaued after about 15 s. In the elderly group, the tasks with auditory information and without external information in Broadmann’s area 10 showed a sudden increase in CBF, which started decreasing at about 15 s after task onset. Contrastingly, similarly aged participants in the implant and elderly groups showed a smaller tendency to increase than did those in the young group. However, there was a tendency toward a slight and monotonic increase.

**Figure 3:**
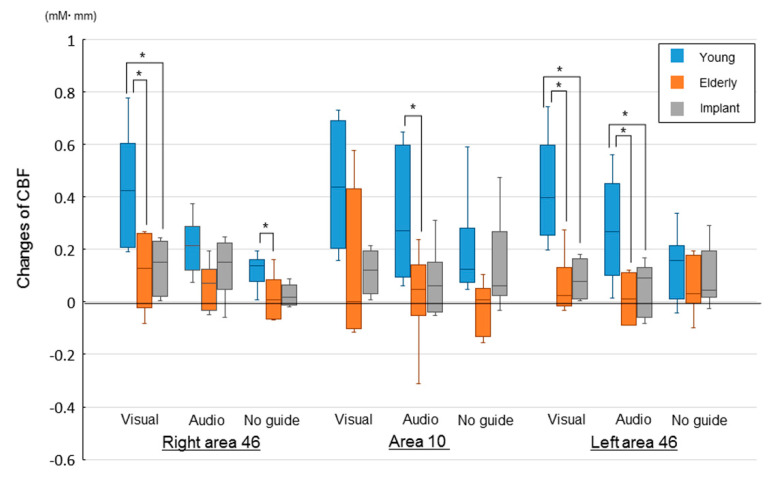
Changes in regional cerebral blood flow of the prefrontal cortex (CBF-PFC) in three groups: visual, with visual information; audio, with auditory information; and no guide, without external information (*p < 0.05).

Figure 3 presents regional CBF changes in each group upon task completion. The young group showed the highest change in every area; moreover, it had the highest cerebral flow in both areas 10 and 46 in the task with the visual information. Compared with the young group, the elderly group showed significantly lower CBF in the right area 46, area 10, and left area 46 in tasks with visual information and without external information, tasks with auditory information, and tasks with visual and auditory information, respectively. Further, these groups showed the greatest decrease in CBF in tasks without external information. Contrastingly, compared with the young group, the implant group showed significantly lower CBF in the right and left area 46 in tasks with visual information and those with visual and auditory information, respectively. There were no significant differences between the elderly and implant groups.

**Figure 4:**
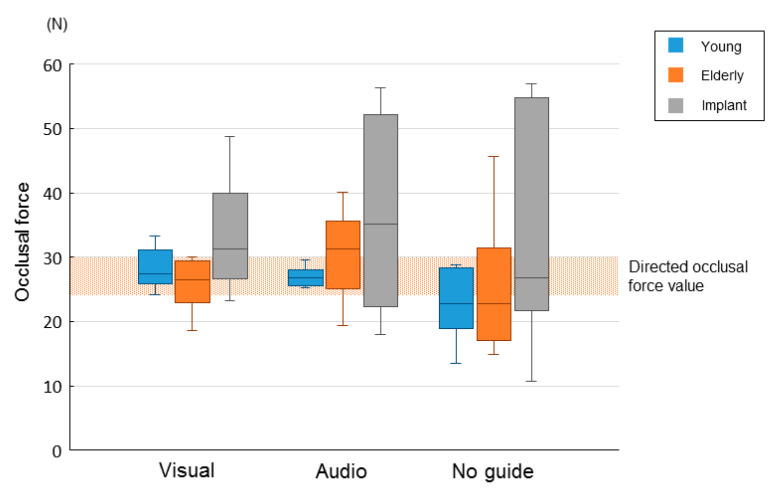
The mean occlusal force value for 20 s, from 10 s after task onset until the end of the task: visual, with visual information; audio, with auditory information; and no guide, without external information.

Figure 4 shows the mean occlusal force value from 10 to 30 s after task onset in all groups. Compared with the young group and the elderly group, the implant group had a numerically, but not significantly, larger occlusal force exceeding the directed range.

**Figure 5:**
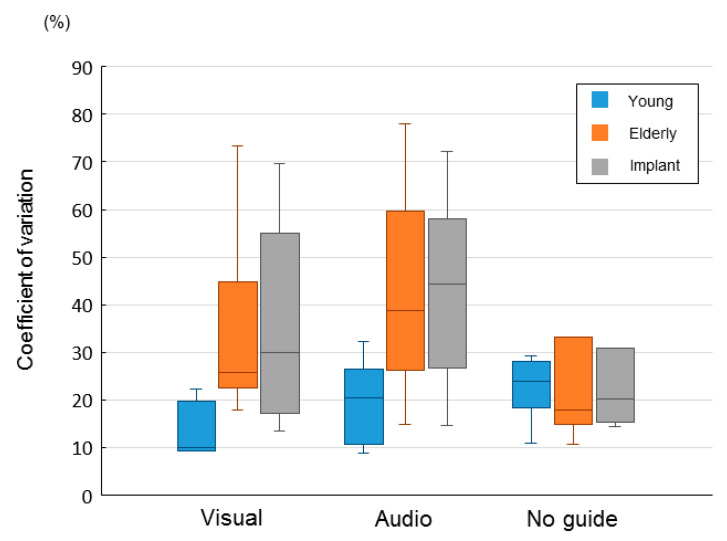
The coefficient of variation of occlusal force for 20 s, from 10 s after task onset until the end of the task: visual, with visual information; audio, with auditory information; and no guide, without external information.

Figure 5 shows the coefficients of variation of the occlusal force in all the groups. The coefficient of variation for the task without external information did not significantly differ among the young, elderly, and implant groups. Regarding the tasks with auditory and visual information, the elderly and implant groups showed higher coefficients of variation than did the younger group; however, there were no significant differences between these groups.

## 4. Discussion

To clarify the effect of periodontal tactile input on the PFC, we used three test groups. It was difficult to find young individuals with implant treatment after tooth loss; therefore, we did not examine this group. Implants lack the pressure-sensing receptors found in teeth, with their role being replicated by sensory receptors in bone tissue and muscle spindles of the closed muscle [14,15,16]. Consequently, the sensory threshold of implants is known to be higher than that of natural teeth with periodontal sensation [17,18]. Concomitantly, we observed an increasing tendency in the occlusal force in the implant group with stability being low. Previous reports indicate that local anesthesia can block periodontal sensation in elderly individuals with natural teeth [19]. However, since it causes physical burden to the elderly and could affect brain function, we did not assess it.

CBF increased in the young, but not the elderly, group during the occlusal force maintenance task. This could be attributed to the effect of decreased PFC activity, learning effect of motor tasks, or reduced sensory afferent information in the periodontal ligament. Regarding decreased PFC activity, aging-related brain function deterioration is considered an inevitable biological phenomenon [20]. Specifically, the PFC is known to be easily affected involving age-related deterioration of executive function, including cognitive-behavioral planning or decision-making [21,22]. Regarding the learning effect of motor tasks, the cerebral cortex, including the PFC, forms multiple loops with the basal ganglia and is involved in motor learning [23,24]. In the early learning stage, with respect to visual perception, the prefrontal loop, which projects from the PFC or parietal lobe cortex via the caudate nucleus, processes visual information and outputs movement signals. When the motor task is encoded and the control is repeated, the motor output is replaced by the motor loop, which projects to the supplemental motor cortex via the putamen and not the PFC. However, even young individuals engage in well-learned masticatory movement; therefore, this mechanism is unlikely. Regarding reduced sensory afferent information in the periodontal ligament, there have been reports of a decline in sensory function with aging, which has been associated with CBF in the PFC [22,25]. In the young group, CBF decreased due to blocked periodontal tactile input by local anesthesia, i.e., decreased periodontal afferent information [9]. In our study, the elderly and implant group had decreased cerebral blood while maintaining occlusal force in the motor task; however, it did not significantly differ with the younger group. This indicates that although periodontal afferent information reduces with age, the motor task could be achieved using other sensory information, including the temporomandibular joint and muscle spindle.

Our findings suggest that periodontal tactile input and the existence of attention behavior function are necessary for increasing CBF in the PFC by inputting periodontal sensation in the task for maintaining occlusal force. During sensory integration, for better sensory signal processing, the PFC outputs processed “top-down” signals to the downstream region [26,27]. Ninomiya reported that multisynaptic frontal inputs, i.e., top-down signals, arise from area 46 to the middle temporal area and visual area 4 [28]. Primate studies have shown that visual area four processes visual guidance, including motion, depth, contrast, and brightness discrimination. Moreover, there is top-down processing of auditory guidance, which is indicated by phenomena such as the cocktail party effect [29,30]. This phenomenon involves focusing on a particular stimulus among a cacophony of conversations or background noise. With respect to PFC activity, it is necessary to consider the existence of top-down signals in such advanced sensory information processing. In the young group, there was increased CBF, which suggests the presence of a unique signal in the PFC during sensory integration. However, in the elderly group, there were no significant differences in the cerebral flow in tasks with/without external information.

The present study has limitations in terms of the different sample sizes of male and female participants. Although the sample size was the same in the implant group, the numbers of male subjects in the young group and the elderly group were five and one, respectively. This might have led to a sampling bias in our study. Goldberg reported that neuropsychological examinations revealed differences regarding prefrontal cortex activity depending on the sex of the participants [31]. It is thus necessary to consider this point when interpreting the results of our study. This study provides the implications of periodontal tactile input and PFC activity. However, in order to clarify the relationship between tooth loss and cognitive decline/dementia, evaluation of more detailed occlusal relations of each participant in this study design and biological approach is needed in future study.

## 5. Conclusions

In conclusion, this study implies that decreased PFC activity or an aging-related decrease in periodontal afferent information did not attribute to the increased CBF in the elderly and implant groups. However, the task of maintaining occlusal force in these groups was similar to that in the young group with the application of afferent information other than periodontal sensation, including the temporomandibular joint and muscle spindle. This suggests that the periodontal tactile input does not uniquely increase PFC activity. However, increased CBF in the PFC due to the periodontal tactile input in the posterior region requires existing attention behavior function in the PFC.
